# Development of a sustainable strategy for cultured fat production based on serum-free 3D culture of bovine adipose stem cells

**DOI:** 10.1038/s41598-025-28441-4

**Published:** 2025-12-29

**Authors:** Zongzhe Xuan, Qiuyue Peng, Mariia Borsuk, Rupali Prasad, Vladimir Zachar, Suman Kumar Das, Cristian Pablo Pennisi

**Affiliations:** 1 Regenerative Medicine Group, Department of Health Science and Technology, Aalborg University, Selma Lagerløfs vej 249, Gistrup, 9260 Denmark; 2 Mirai International AG, Herdernstrasse 71, Zurich, 8004 Switzerland

**Keywords:** Cultured fat, Serum-free medium, 3D spheroid culture, Adipogenic differentiation, Bovine adipose stem cells, Sustainable meat production, Stem-cell biotechnology, Tissue engineering, Lipids

## Abstract

Cultivated meat provides a sustainable and ethical alternative to traditional livestock production, addressing global challenges in meat demand and environmental impact. Fat is recognized as an essential component of meat products, but strategies for culturing animal fat precursors still have major limitations, such as the need for a serum-containing medium and inefficient adipogenic cell maturation. In this study, a serum-free medium (SFM) formulation for the cultivation of bovine adipose-derived stem cells (bASC) was first validated by comparison with serum-containing media (FBS). After a 14-day induction period, the SFM increased bASC proliferation by 20% and triglyceride accumulation by 66%. When the SFM was applied to a three-dimensional (3D) spheroid culture system, it resulted in up to 34% higher triglyceride accumulation compared to 2D cultures. These results were accompanied by a significant increase in the expression of key adipogenic markers and lipid droplet formation, suggesting a synergistic potential of SFM and 3D spheroid strategies for bASC culture. Overall, the results of this study provide a scalable and sustainable platform for cultured fat production and are an important step towards overcoming some of the challenges in cultured meat production.

**Supplementary Information:**

The online version contains supplementary material available at 10.1038/s41598-025-28441-4.

## Introduction

Cultured or cultivated meat, produced from animal cells using tissue engineering methods, has gained significant attention in recent years due to its potential to offer a more ethical and sustainable alternative to conventional meat production^1,2^. Unlike plant-based meat substitutes, cultivated meat aims to replicate the sensory experience of traditional meat, including its texture, taste, and appearance^3^. While much of the research in cultivated meat focuses on muscle tissue as the primary protein source, the role of fat in meat cannot be overlooked. Fat contributes to the juiciness, flavor, and mouthfeel of meat, which are critical for consumer acceptance^4^. During cooking, for example, meat releases hundreds of volatile compounds, most of which originate from fats^5^. Additionally, fat is essential for creating the species-specific flavor that distinguishes the taste of different meats^6^. Therefore, producing and integrating tissue engineered fats into cultivated meat products is vital for achieving a product that meets consumer expectations^3^.

In vivo, the lipids that contribute to the sensory properties of meat are primarily synthesized and stored within adipocytes, specialized cells that form adipose tissue. Adipocytes play a crucial role in lipid metabolism, through synthesis, accumulation, and release of fatty acids that are essential for the development and function of adipose tissue^7^. Adipose stromal/stem cells (ASCs) have been identified as a valuable source for generating adipocytes in vitro due to their inherent ability to differentiate into lipid-producing cells^8,9^. To support the growth and differentiation of ASCs in culture media are supplemented with fetal bovine serum (FBS), which provides the necessary growth factors and nutrients to promote efficient proliferation and adipogenic differentiation of the cells^10,11^. However, the cost of using culture and differentiation media containing FBS is a major limiting factor in scaling up production of cultivated meat^12^. Additionally, the reliance on animal-derived FBS raises ethical concerns and limits the sustainability of the process, conflicting with the overarching goals of cultivated meat production. With the aim of eliminating dependence on products of animal origin, while ensuring efficient cell growth and differentiation, the development of serum-free alternatives has been extensively studied in the context of expanding ASCs for clinical applications^13–15^. In the field of culture meat, serum-free media formulations are crucial for the development of a sustainable alternative to conventional meat. The majority of efforts have so far focused on optimizing media for muscle precursor cells, likely due to their central role in defining the texture and protein content of cultured meat products^16–18^. In contrast, serum-free strategies tailored specifically for bovine fat cells remain relatively underexplored, but recent studies have begun to address this gap. Mitić et al. introduced a simplified, cross-species serum-free formulation capable of supporting adipogenic differentiation^19^. Similarly, Messmer et al. applied single-cell transcriptomic analysis to identify bovine muscle-derived cell subtypes with adipogenic potential, providing insights for more targeted media development^20^. More recently, refinement of media composition has focused on replacing traditional components like glutamine and glucose with alternatives that minimize toxic byproducts such as ammonia, thereby enhancing both cell proliferation and adipogenic differentiation^21^.

Traditionally, ASCs are cultured in two-dimensional (2D) monolayer systems, which do not accurately mimic the complex microenvironment of tissues^22^. Additionally, prolonged culture of ASCs in 2D conditions often results in a loss of replicative ability, colony-forming efficiency, and differentiation capacity^23^. A promising alternative is the cultivation of ASCs as three-dimensional (3D) tissue structures that better mimic the natural stem cell microenvironment^24–26^. Research with human ASCs has shown that 3D spheroids represent a more relevant system for the differentiation and maturation of the cells into the adipogenic lineage, producing cells that are more similar to mature adipocytes^27–29^. However, protocols developed for adipogenic differentiation of human and mouse cells showed low efficiency in inducing adipogenesis of bovine progenitor cells, emphasizing that there are significant differences between human and ruminant adipogenic processes^30^. For instance, human cells exhibit higher enzymatic activity for converting glucose to fatty acids, while bovine cells rely more on acetate due to their unique metabolic adaptations^31^. Furthermore, while more recent studies have begun to understand the effect of 3D culture in bovine adipogenic precursors^30,32^, these studies were conducted in serum-containing media. Studies have shown that human mesenchymal stromal cells form loose 3D aggregates but not solid spheroids after serum withdrawal^33^, which could be related to removal of the adhesion proteins contained in the medium, including fibronectin and vitronectin^34^. Therefore, it is not yet known whether the removal of serum influences the 3D cultivation of bovine ASCs. In this study, we address these challenges by comparing 2D and 3D culture methods for bovine ASCs, focusing on adipogenic differentiation in serum-free media. By characterizing these properties, we aim to provide a foundation for the efficient and sustainable production of cultivated fat, potentially paving the way for large-scale adoption of these methods in cultivated meat production.

## Results

Initially, the mesenchymal identity of the isolated cells was assessed through transcriptional and flow cytometric analyses. RT-qPCR revealed positive expression of genes encoding for mesenchymal markers CD29, CD44, CD73, and CD166, while the gene encoding for the hematopoietic marker CD45 was absent (Figure S1, Supplementary information file). Flow cytometry further supported this identity, showing surface expression of CD44 (99.9%), and CD90 (33.3%), but minimal CD45 signal (2.6%) (Figure S2, Supplementary information file). These findings collectively confirm the mesenchymal origin of the isolated bovine adipose-derived stem cells (bASCs). The isolated bASCs were also characterized using colony-forming unit–fibroblastic (CFU-F) to assess self-renewal and clonogenic capacity. Clones were consistently formed at all seeding densities tested, demonstrating the self-renewal and proliferation capacity of the bASCs (Figure S3, Supplementary information file).

**Fig. 1:**
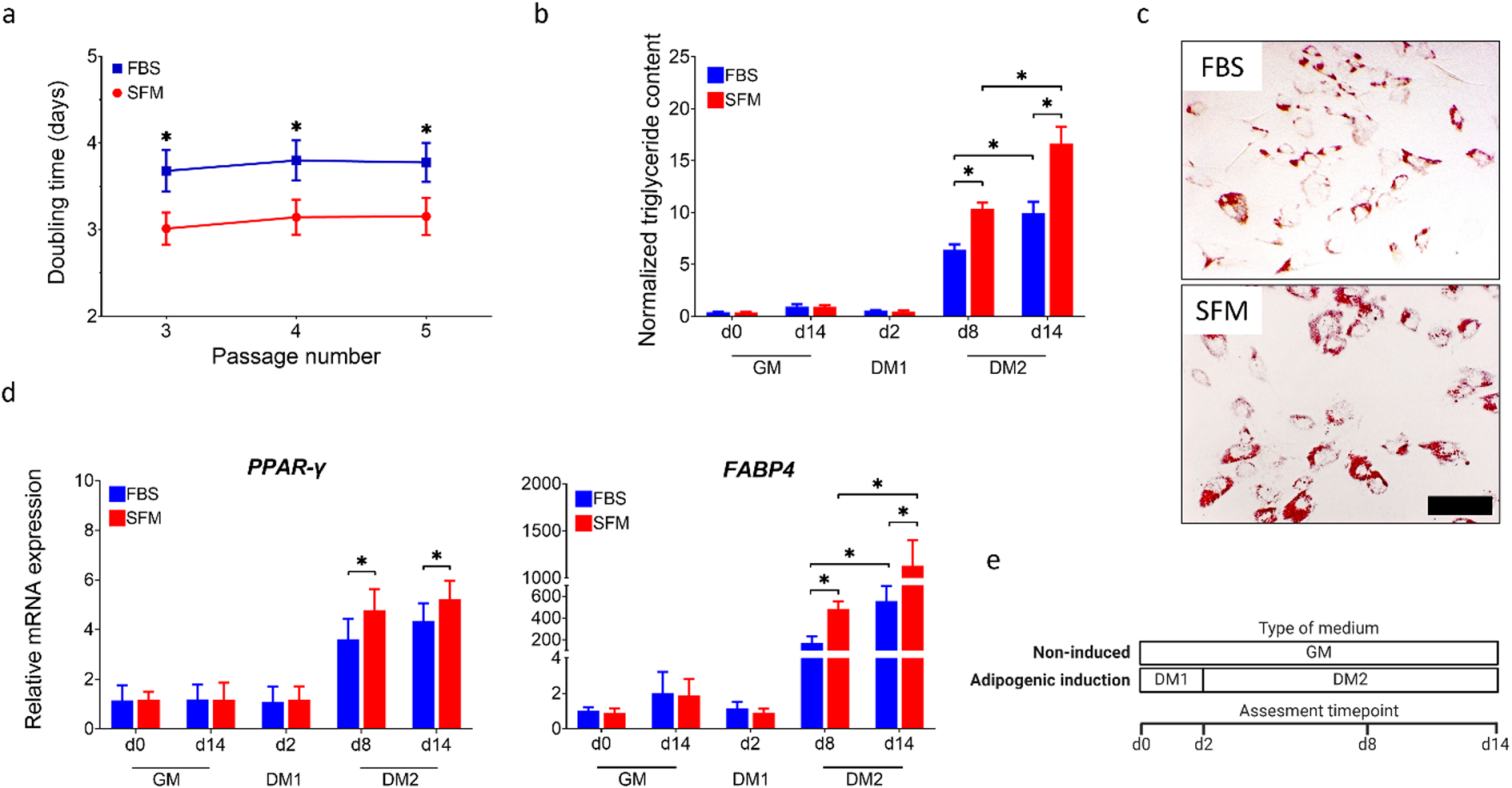
Growth and adipogenic differentiation of bASCs in serum-free medium (SFM) and serum-containing medium (FBS). (a) Doubling time of cells at passages 3, 4, and 5. (b) Triglyceride accumulation normalized to cell number at different timepoints post-induction in growth medium (GM), differentiation medium for induction (DM1), and differentiation medium for maintenance (DM2). (c) Representative images of Oil Red O staining showing the formation of lipid droplets in bASCs at day 14 of adipogenic induction (scale bar = 100 μm). (d) Relative mRNA expression levels of adipogenic markers, peroxisome proliferator-activated receptor gamma (*PPAR-γ*) and fatty acid-binding protein 4 (*FABP4*), measured by RT-qPCR at different time points in GM, DM1, and DM2. (e) The diagram shows the assessment time points (d0, d2, d8 and d14) and the type of medium used in the induced and non-induced groups. Data are expressed as mean ± SEM (* *p* < 0.05).

The growth and differentiation potential of the bASCs was investigated under serum-free conditions. Over passages 3 to 5, bASCs cultured in serum-free medium (SFM) showed a significantly higher proliferation capacity than those cultured in serum-containing medium (FBS). As shown in Fig. 1, the doubling time of cells in SFM was significantly shorter, indicating a faster proliferation rate in the absence of serum. The percentage increase was approximately 20%. In addition, Ki-67 analysis revealed significantly higher expression of this proliferation marker in cells cultured in SFM after prolonged passage, further supporting the sustained proliferation potential of bASCs under these conditions (Figure S4, Supplementary information file). Non-induced controls were maintained in growth medium (GM) for the duration of the experiment, while differentiation was induced using a two-step protocol consisting of induction medium (DM1) followed by maintenance medium (DM2). After initiation of adipogenic differentiation, bASCs in SFM exhibited a substantial increase in intracellular triglyceride accumulation, as shown in Fig. 1. Compared to bASCs cultured in FBS, cells in SFM displayed significantly higher triglyceride content on days 8 and 14, indicating a more robust differentiation response. On day 14, when comparing the mean values of normalized triglyceride content (16.62 in SFM vs. 9.96 in FBS), it can be inferred that the percentage increase in lipid accumulation due to SFM was over 66%. This enhanced lipid accumulation was also confirmed by Oil Red O staining on day 14 (Fig. 1), in which cells cultured in SFM showed more intense staining of lipid droplets compared to their FBS counterparts. At the transcriptional level, adipogenic differentiation in SFM was further supported by the expression profiles of key adipogenic markers, *PPAR-γ* and *FABP4*. As shown in Fig. 1, while only minimal changes in gene expression were observed after 2 days of induction (DM1), cells cultured in SFM exhibited significant upregulation of both markers at later stages of differentiation in DM2 (day 8 and 14). On day 14, the expression of *PPAR-γ* and *FABP4* was significantly higher in bASCs cultured in SFM than in FBS.

**Fig. 2:**
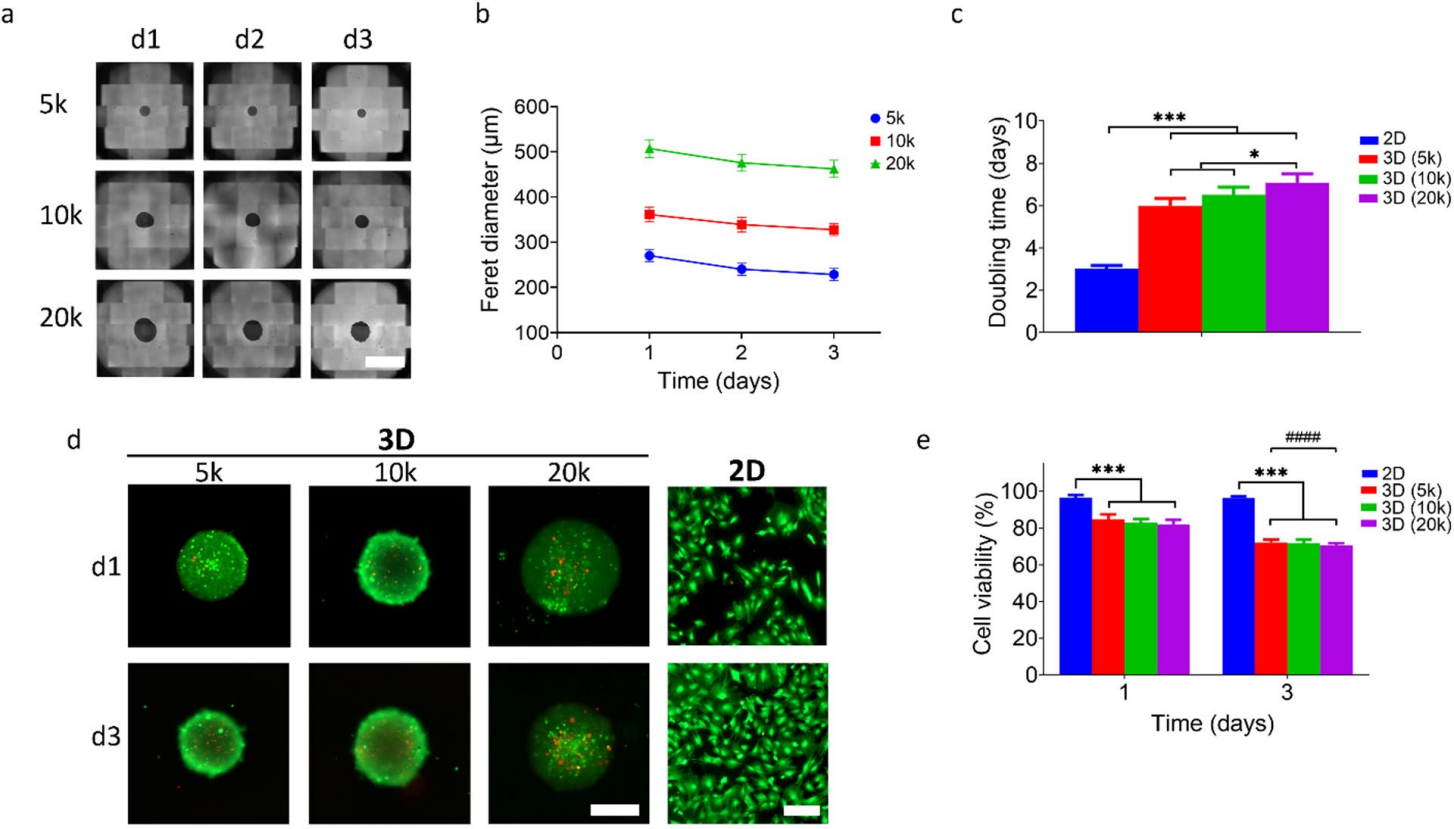
Morphology and viability of spheroids formed at different initial densities. (a) Representative phase-contrast microscopy images showing the morphological development of bASC spheroids. The spheroids were formed from three different initial seeding densities: 5000, 10,000, or 20,000 cells/well. The images were taken over three days of culture, labeled as d1, d2 and d3. Scale bar = 1 mm (b) Quantification of spheroid size for each seeding density. (c) Comparison of doubling times between 2D monolayer cultures and 3D spheroids. (d) Live/dead staining of bASCs cultured in 2D monolayers and 3D spheroids. Live cells are stained green, and dead cells are stained red (scale bar = 200 μm (3D) & 100 μm (2D)). (e) Quantitative analysis of cell viability across 2D and 3D cultures. Data are presented as mean ± SEM (* *p* < 0.05, ^# # # &^ *** *p* < 0.001, ^#^ Represents the comparative analysis between d3 and d1).

The morphology and viability of spheroids formed from bASCs seeded at different initial densities were analyzed over a three-day culture period using SFM. Figure 2 illustrates the self-organization of cells into spheroids at different densities (5000, 10000, and 20000 cells per well) from day 1 to day 3. On day 1 after seeding, the spheroids exhibited a quasi-circular morphology characterized by well-defined edges, with almost all seeded cells seamlessly integrated into the spheroid structure. A clear seeding density-dependent morphological difference was observed, with higher initial cell densities leading to larger spheroids. Quantitative analysis of spheroid size, represented as Feret diameter, confirmed that spheroid size was directly correlated with initial cell density (Fig. 2). The doubling time of bASCs cultured in 3D spheroids was compared with different cell densities and with 2D monolayer cultures (Fig. 2). Cells cultured under 2D conditions showed a significantly shorter doubling time than cells in 3D spheroids, with the doubling time increasing significantly with increasing initial cell density in the spheroids. Cell viability was assessed using live/dead staining. Figure 2 shows representative images of both 3D spheroids and 2D monolayer cultures on days 1 and 3, with live cells shown in green and dead cells in red. In 3D cultures, the spheroids formed at higher densities (10k and 20k) exhibited more pronounced red staining, indicating a higher degree of cell death over time. In contrast, the 2D cultures maintained a higher overall viability over the same period. Quantitative analysis of cell viability confirmed the microscopy results, with cells in all 3D conditions consistently exhibiting lower viability compared to 2D cultures (Fig. 2). All three spheroid groups with different seeding densities appeared to have relatively high cell viability on day 1 (> 80%). Cell viability in 3D spheroids decreased significantly from day 1 to day 3, but there was no statistical difference in cell viability between the different seeding densities. Statistical analysis confirmed the significant differences in viability between 2D and 3D cultures and between day 1 and day 3 for the 3D conditions (### *p* < 0.001). Representative flow cytometry plots used for this quantification are provided in the supplementary information file (Figure S5).

**Fig. 3:**
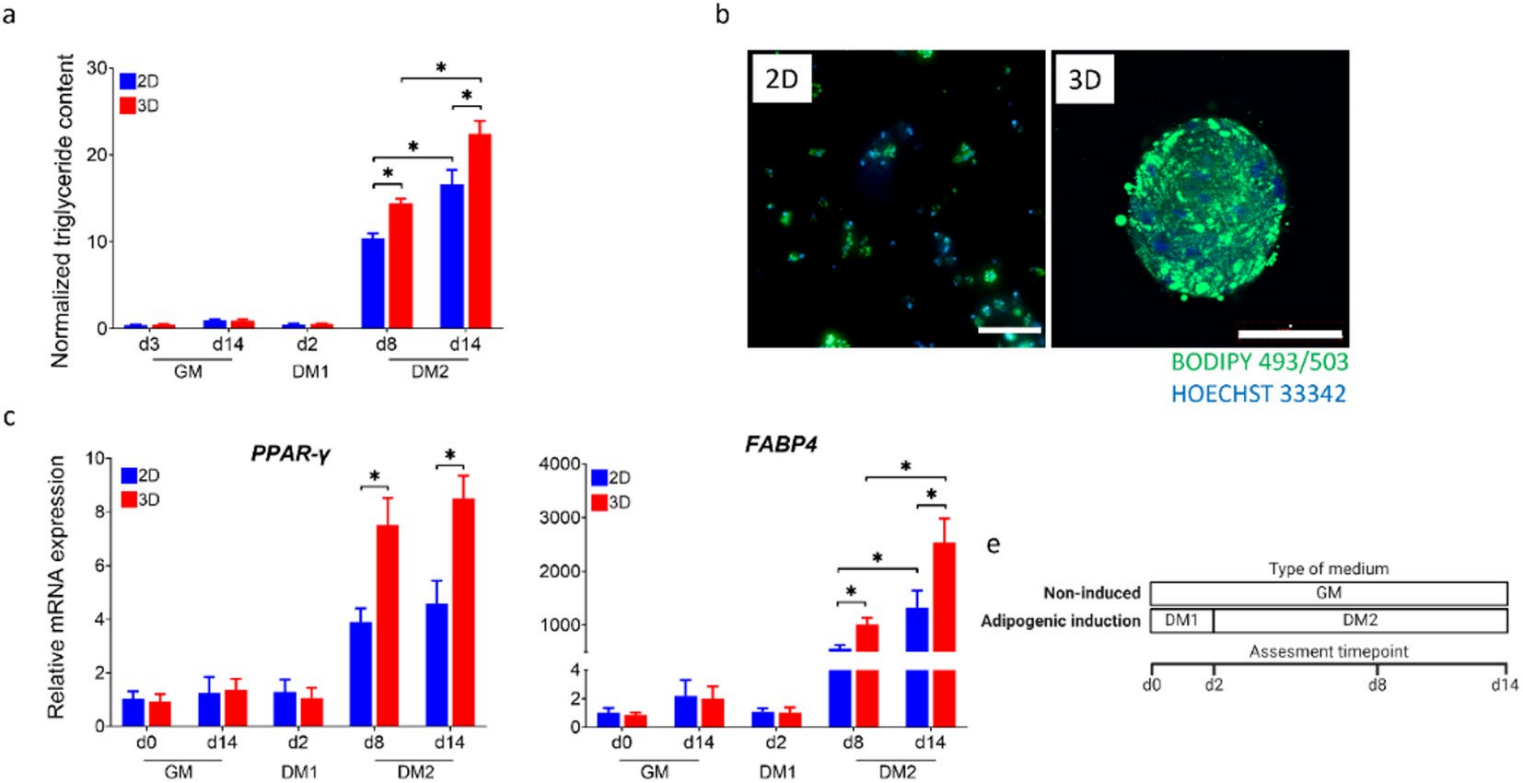
Adipogenic differentiation of bASCs in 3D spheroids and 2D monolayer cultures. (a) Normalized triglyceride content measured at different time points in serum-free growth medium (GM), differentiation medium for induction (DM1), and differentiation medium for maintenance (DM2). (b) Representative images of BODIPY 493/503 staining showing lipid accumulation (green) in bASCs cultured in 2D and 3D after 14 days of adipogenic induction. Cell nuclei counterstained with Hoechst 33,342 (in blue) (scale bar = 100 μm). (c) Relative mRNA expression levels of adipogenic markers, peroxisome proliferator-activated receptor gamma (*PPAR-γ*) and fatty acid-binding protein 4 (*FABP4*), measured by RT-qPCR at different time points in GM, DM1, and DM2. (e) The diagram shows the assessment time points (d0, d2, d8 and d14) and the type of medium used in the induced and non-induced groups. Data are expressed as mean ± SEM (* *p* < 0.05).

The adipogenic differentiation potential of bASCs was investigated under 3D spheroid culture conditions using SFM, with 2D monolayers as reference. As shown in Fig. 3, adipogenic induction in 3D spheroids led to a significant increase in intracellular triglyceride content. After 8 days in DM2, the spheroids showed significantly higher triglyceride levels compared to the early induction phase (DM1) and the non-induced controls (GM). On day 14, the spheroids showed the highest triglyceride accumulation compared to the conventional 2D cultures. The percentage increase in triglyceride accumulation in 3D was over 34%. Further confirmation of adipogenic differentiation was provided by BODIPY 493/503 staining, which demonstrated the formation of lipid droplets. At 14 days post-induction, bASCs in 3D spheroids exhibited extensive lipid accumulation, with significantly greater lipid droplet coverage compared to 2D monolayers (Fig. 3). The 3D spheroid cultures exhibited a dense and organized distribution of lipid droplets, indicating a more mature adipogenic state. At the transcriptional level, after 8 days in DM2, the expression of *PPAR-γ* and *FABP4* was significantly increased in the 3D culture compared to early differentiation stages and 2D monolayer culture (Fig. 3). At day 14, bASCs in 3D spheroids continued to exhibit much higher expression of *PPAR-γ* and *FABP4* than their 2D counterparts, consistent with the increased lipid accumulation observed under 3D conditions.

## Discussion

While most in vitro adipogenesis studies in the past have been based on serum-containing media, the focus in the cultured meat field has been on the development of serum-free media suitable for the expansion and differentiation of progenitor cells^2^. The use of FBS is associated with several challenges, including ethical concerns regarding animal welfare, risk of pathogen contamination, unpredictable supply and high costs^35^. Therefore, development and optimization of serum-free media is critical to achieve the scalability and sustainability required for large-scale cultured meat production^36,37^. The challenges associated with formulating serum-free media have been extensively addressed in previous research, with significant progress made in creating chemically defined media to support the differentiation of various progenitor cells, including bovine satellite cells^16,18^ and adipogenic progenitor cells from several species^19^. In this study, the initial focus is on the validation of existing serum-free culture media for the expansion and differentiation of bASCs, with the main objective being to ensure that the media can support the culture of the cells under well-defined conditions. This validation step is critical to assess the viability of 3D culture conditions and provide a basis for future scalability and sustainability in the context of cultured meat production.

In terms of cell proliferation, the SFM was more efficient than serum-containing media, as evidenced by a reduced doubling time and relatively higher expression of the proliferation marker Ki-67. This cell cycle-associated protein is expressed during the active phases of the cell cycle (G1-M), so an increased expression is an indication of active cell proliferation^38^. These findings highlight the validity of the SFM not only to replace FBS but also to enhance cell growth performance. Previous studies have investigated the role of serum in the proliferation and differentiation of bovine adipogenic progenitor cells, particularly muscle-derived fibroadipogenic progenitors (FAPs). While these cells differentiate efficiently under serum-free conditions, their expansion phase is normally carried out in serum-containing medium^39^. Notably, FAPs cultured without serum show comparable gene expression profiles to cells grown under serum-containing conditions^20^. Recent efforts have aimed to simplify adipogenesis protocols by reducing the number of inducers and differentiation phases to facilitate upscaling^19^. For the differentiation phase, the approach in this study incorporated two distinct serum-free media: one specifically formulated for adipogenic induction and the other for maintenance. This dual-media system effectively supported both the early stages of adipocyte commitment and the later stages of lipid accumulation, as evidenced by higher expression levels of key adipogenic transcription factors, such as *PPARγ* and *FABP4*. These transcription factors play a critical role in preadipocyte determination^40,41^. The separation of induction and maintenance phases allows for optimal regulation of the differentiation process, maximizing the efficiency of lipid accumulation in the absence of serum-derived growth factors, which have been shown to influence adipogenic outcomes unpredictably^19^. Studies on human ASCs demonstrated that using defined media in each phase (differentiation and long-term maintenance) could enhance cell adherence and lipid accumulation while maintaining cell functionality over extended culture periods^42^.

Studies have shown that 3D culture conditions enhance the production of mature adipocytes from human and murine precursors^27–29^. However, bovine adipogenic models face unique challenges, including an often inconsistent adipogenic differentiation compared to other species, which makes the validation of 3D culture conditions particularly critical in the bovine context^30^. The results of this study show that bASCs rapidly formed stable spheroids within three days at all cell densities tested. The diameter of the spheroids decreased after their initial formation, which correlated with the density of cells seeded in each well and the incubation time. These observations are consistent with previous studies that have shown that the size of cell spheroids can be adjusted by controlling cell seeding density^43^. As in previous studies, the viability of bASCs decreased significantly under 3D spheroid culture conditions compared to 2D culture throughout the process of spheroid formation^44^. For spheroids in the diameter range between 200 and 500 μm, studies have shown that there is a gradual accumulation of metabolic waste and insufficient diffusion of oxygen/nutrients, which could be the main reason for the decrease in cellular viability^45^.

Following the assessment of viability and growth, the results show that the 3D spheroid system provided an enhanced microenvironment for adipogenesis of bASCs, as evidenced by the increase in triglyceride content and larger lipid droplets within the spheroids after adipogenic induction. The expression of transcription factors *PPARγ* and *FABP4* was consistently higher in spheroid cultures than in 2D monolayers, leading to more mature adipocytes with larger lipid droplets. The 3D environment may mimic aspects of the native extracellular matrix, offering a more physiologically relevant context that encourages adipogenic commitment and maturation^46^. Furthermore, low oxygen (hypoxic) conditions are known to modulate ASC behavior, particularly through enhanced proliferation and increased secretion of growth factors that can further promote differentiation^15,44,47^. Hypoxia and cell morphology can influence adipogenic differentiation, as shown by the improved adipogenic outcomes in ASCs when cytoskeletal tension and oxygen levels are reduced^48^. Thus, a combination of reduced cytoskeletal stress and limited oxygen diffusion within the dense spheroids may also have contributed to the increased adipogenic efficiency observed in 3D. The efficiency of adipogenic differentiation of bASCs is particularly important for cultured fat production^49^. Notably, the use of serum-free media in the 3D system proved critical for achieving this level of adipogenic maturity, further underscoring the advantages of 3D cultures over conventional 2D systems.

While the presented 3D approach is effective for cell differentiation, large-scale production of cultured fat requires extensive cell expansion prior to the differentiation phase to generate substantial amounts of cellular material from limited donor tissue^50^. Although the 3D spheroid format supports adipogenic maturation, our data indicated that it is not optimal for cell expansion. Our viability assays indicated a gradual decline in cell viability over time, particularly in dense spheroids, consistent with diffusion limitations observed in similar systems. Studies have shown that in such constructs, nutrient and oxygen gradients can develop, leading to reduced proliferative activity and compromised cell survival in the spheroid core^51,52^. These intrinsic limitations reinforce the need for complementary expansion systems to achieve scalable production of cultured fat. A two-step strategy may be required, such as initial expansion in bioreactors (e.g., suspension or hollow fiber systems), followed by differentiation in 3D. Additionally, strategies to improve mass transport within 3D constructs, such as dynamic culture systems, perfusion bioreactors, or carrier-supported formats, have shown promise in mitigating these limitations and enhancing viability^49^.

In addition, the reliability and reproducibility of stem cell sources are crucial^53^. In this study, the cells were isolated according to a standard protocol, which generally provides very consistent results in terms of yield of ASCs. Their mesenchymal identity was confirmed through transcriptional profiling and flow cytometric analysis, which revealed expression of typical MSC markers and absence of the hematopoietic marker CD45. These findings are consistent with previous characterizations of bovine MSCs, in which there is consensus for positive expression of CD44, negative expression of CD45, while expression of other markers has shown variability depending on the cell isolation method and antibody source^54–59^. To further assess stemness, colony-forming unit–fibroblastic (CFU-F) and osteogenic differentiation assays were performed. The cells demonstrated robust self-renewal and proliferation capacity, forming clones across all tested seeding densities. Notably, even when the cells were seeded sparsely, the clonogenic efficiency was higher than previously reported^54^. Importantly, the maintenance of multipotency after passaging was confirmed by differentiation assays where the cells retained both osteogenic and adipogenic potential after 9 passages (Figure S6 and S7, Supplementary information file), supporting their suitability as seed cells for scalable cultured fat production. One possible limitation is the fact that only cells from one donor were used. To ensure the robustness and reproducibility of the findings, future research should validate these results with cells from multiple animal donors and understand the behavior of the cells in long-term cultures, which may impact the efficiency of adipogenic differentiation and scalability for cultured fat production. In human ASCs, significant immunophenotypical and biological changes occur during in vitro expansion, underscoring the importance of monitoring cell behavior over extended culture periods^60^.

In summary, this study presents a novel method for culturing bASCs into mature adipocytes in a 3D spheroid culture system under serum-free conditions. Compared to conventional 2D cultures in serum-containing media, the presented method enables efficient lipid accumulation. These results indicate the potential of this approach for cultured fat production. However, further validation with different cell donors is required to ensure the robustness and reproducibility of the protocol. Overall, this research contributes to the advancement of cultured meat technologies by addressing critical challenges in the cell maturation phase, potentially paving the way for large-scale adoption of these methods in cultured meat production.

## Materials and methods

### Isolation and culture of cells

Primary bovine ASCs (bASCs) were isolated from the subcutaneous adipose tissue of 6-month-old Black Angus calf (collected at the abattoir) following previously described protocols^61,62^, which were adapted for processing excised tissues. As the material was obtained from a deceased animal as a by-product of a routine commercial practice, no ethical approval was required. This is in accordance with Directive 2010/63/EU on the protection of animals used for scientific purposes. In brief, tissue samples were transferred to a sterile hood and disinfected in 70% ethanol for 2 min. The samples were washed with PBS and subsequently excised from visible connective, muscle tissue and blood vessels in isolation medium comprising Dulbecco’s modified Eagle medium (DMEM) supplemented with 10% fetal calf serum (FBS), 12.5 µg/ml amphotericin B, and 500 U/ml penicillin/streptomycin (all from ThermoFisher Scientific). The part of the tissue containing only white fat cells was minced into small pieces (~ 2 mm^3^) in isolation medium using sterile forceps and scissors. The tissue pieces were then digested with STEMxyme 1 solution (BioConcept Ltd, Switzerland) in a shaking incubator/water bath at 37 °C for 2 h. The resulting mixture was centrifuged at 80 g for 5 min at 4 °C and the supernatant was filtered through cell strainers. The cell suspension was then incubated on ice in erythrocyte lysis buffer for 5 min. After careful washing and resuspension, the cells were plated and expanded in growth medium (GM) consisting of DMEM/F-12 nutrient mixture (DMEM/F-12, Gibco), supplemented with 10% FBS, and 100 U/ml penicillin/streptomycin. The cells were maintained in tissue culture flasks (Greiner Bio-one) in a standard humidified incubator at 37 °C and 5% CO_2_. The culture medium was changed every second day. Cells were passaged using TrypLE select (Gibco, ThermoFisher Scientific) when they reached 90% confluence.

### Surface marker analysis

To characterize the surface marker expression of bASCs, flow cytometry was performed using an antibody panel targeting two mesenchymal and one hematopoietic marker. Specifically, bASCs at passage 3 were stained with CD44 antibodies conjugated with BV421 (cat. no.103040, BioLegend, San Diego, CA, USA), CD90 antibodies conjugated with PE (cat. no. NBP2-47755PE, Novus Biologicals, Centennial, CO, USA) and CD45 antibodies conjugated with FITC (cat. no. MCA832F, BioRad, Hercules, CA, USA). To ensure accurate gating and viability assessment, cells were first incubated with the fixable viability dye 570 (FVS 570, BD Biosciences, Lyngby, Denmark) for 15 min at room temperature. Antibody cocktails were then added and incubated for 30 min at 4 °C in flow staining buffer consisting of 2% fetal calf serum and 0.1% sodium azide in PBS. Fluorescence minus one (FMO) controls were included to define gating boundaries, with the top 2.5 percentile of FMO signal used to set positivity thresholds. The data obtained were visualized and analyzed using the Kaluza 2.1 software package (Beckman Coulter, Indianapolis, IN, USA).

### Evaluation of self-renewal and clonogenic potential

The bASCs were seeded in triplicate in 6-well plates at four different densities (20, 60, 120, and 150 cells/well) and cultured in serum-containing GM for 14 days. The medium was changed on day 7 and every 3 days thereafter. On day 14, the cells were fixed with 4% formaldehyde and stained with 0.05% crystal violet (Sigma-Aldrich). The plates were placed on an LED light pad (Shenzhen Huion Trend Technology) and images were captured with a single-lens reflex camera (EOS750D, Canon). The colony forming units (CFU-F) efficiency was determined in the 60 cells/well group by dividing the initial cell density by the average number of cell colonies formed.

### Preparation of cultures for assessment of cell growth and differentiation

To evaluate the performance of the serum-free media in terms of proliferation and differentiation, bASCs were seeded at a density of 5,000 cells/cm^2^ on standard 6-well plates (Greiner, Bio-One) previously coated with a 0.2% gelatin solution. Once the bASCs were confluent, the cells were differentiated into mature adipocytes in two phases. In the first phase, bASCs were induced into preadipocytes by incubation with the first differentiation medium (DM1) for 48 h. Subsequently, the preadipocytes were maintained in the second differentiation medium (DM2) for 12 days. The non-induced control groups were cultured in GM for the entire assessment period.

Serum-containing DM1 was prepared according to the protocol of Kilroy et al.^63^ with minor modifications. The DMEM/F-12 nutrient mixture was supplemented with 15% FBS, 33 µM biotin, 17 µM pantothenate, 0.2 µM insulin, 1 µM dexamethasone, 0.5 mM isobutylmethylxanthine (IBMX), 5 µM rosiglitazone, and 100 U/ml penicillin/streptomycin. Serum-containing DM2 was prepared according to Mehta et al.^11^ using DMEM/F-12 supplemented with 10% FBS, a mixture of free fatty acids (5 µM pristanic acid, 1 µM phytanic acid, 50 µM myristoleic acid, 5 µM elaidic acid, 5 µM erucic acid, 5 µM oleic acid, and 150 µM palmitoleic acid) and 100 U/ml penicillin/streptomycin. The serum-free GM (FatSFM, Mirai International) consists of DMEM/F12 and supplements. Serum-free DM1 (FatDM1, Mirai International) consists of Glasgow’s modified Eagle medium (GMEM) and supplements. The serum-free DM2 (FatDM1, Mirai International) is based on GMEM, insulin and free fatty acids. The exact composition of the serum-free media is proprietary to Mirai International.

To evaluate the influence of three-dimensional (3D) culture conditions on bASC behavior, experiments were performed exclusively in serum-free media formulations. After culturing the bASCs in serum-free GM in tissue culture flasks for at least 7 days, the cells were trypsinized and seeded on 96-well spheroid plates (BIOFLOAT, faCellitate) at three different densities (5000, 10000 and 20000 cells/well). These plates provide a highly defined cell-repellent surface on which spheroids can be formed without the need for a scaffold. To prevent spheroids from being lost during media changes, only half of the media volume was replaced with each media change. The control group comprised cells cultured in 6-well plates treated in the same way for consistency. The process of spheroid formation was monitored at different time points using a plate scanner with digital phase contrast (CytoSMART Omni, Axion). The device was set to scan the spheroids every six hours for 72 h. The images were collected and then analyzed using Image J to quantify the process of spheroid change over time and compare the size of the different seeding densities.

### Proliferation assay

To evaluate cell proliferation under different culture conditions, a Presto Blue assay (Invitrogen, Thermo Fisher Scientific) was performed with cells between the third and fifth passage. After 24, 48, 72 and 96 h, Presto Blue reagent was added to the cell-containing wells at 10% of the total volume, followed by incubation at 37 °C for 3 h. The negative controls consisted of cell-free wells. Subsequently, 100 µl of the solution was loaded (in duplicates) in a 96-well microplate. Fluorescence was measured using a multimode plate reader (EnSpire, PerkinElmer) at excitation and emission wavelengths of 560 nm and 610 nm, respectively. After fluorescence measurement, the cells were washed with PBS and fresh growth medium was added. Cell proliferation was expressed as doubling time based on the actual measurement of fluorescence intensity (*n* = 6), as previously reported^64^.

### Cell viability assessment

To assess cell viability, live and dead cells were stained 1 and 3 days after seeding. Cells were first washed with PBS and incubated for 1 h in a serum-free DMEM/F-12 nutrient mixture containing calcein-AM (1 µM) and propidium iodide (5 µM). The cells were then washed twice with serum-free DMEM/F-12. Samples were observed using a fluorescence microscope (Axio Observer Z1, Carl Zeiss) and images were captured using a digital camera (C11440 ORCA Hamamatsu) and ZEN 2012 software.

Quantitative analysis of cell viability was performed using a flow cytometer (CytoFLEX, Beckman Coulter, Copenhagen, Denmark). The spheroids were harvested through a 40 μm BD Falcon cell strainer and subsequently dissociated with a cell dissociation reagent (StemPro Accutase, Gibco), followed by two washes with PBS. The dissociated cells were resuspended in PBS at a concentration of 1 × 10^6^ cells/100 µl. To quantify the number of live and dead cells, FVS 570 was added to the cell suspensions and incubated at room temperature in the dark for 15 min. Prior to analysis, the cells were washed and resuspended in flow staining buffer. The experiments were repeated three times, each time recording at least 2.5 × 10^4^ individual cells. Data acquisition and analysis were performed using the same instrumentation and software described in the surface marker analysis section.

### Quantification of intracellular triglyceride and DNA content

An adipogenesis test kit (MAK040-1KT, Sigma Aldrich) was used to determine the total cellular concentration of triglycerides. This assay is based on an enzymatic reaction that generates a product that is measured by spectrofluorometry. The assay was performed according to the manufacturer’s instructions. In brief, the lipid extraction reagent was added to the cells in 2D monolayers and 3D spheroids. The spheroids were disrupted by vigorous pipetting. The measurement was performed with a multimode plate reader (EnSpire, PerkinElmer) at excitation and emission wavelengths of 535 nm and 587 nm, respectively. The data were normalized to the DNA content of each sample. The DNA content of each well was measured using the AccuClear dsDNA quantification kit (Biotum) according to the manufacturer’s instructions. In brief, 10 µL of each sample was added to 200 µL of working solution prepared by diluting the dye 100-fold with the DNA quantification buffer and mixed by pipetting. After a 5-minute incubation at room temperature in the dark, the plate was read on a multimode plate reader with excitation and emission of 468 and 507 nm, respectively. The results obtained were fitted to a DNA standard curve to determine the mass of DNA per sample (*n* = 3).

### RNA extraction and quantitative real-time RT-PCR analysis

A quantitative real-time PCR was performed to investigate the transcriptional activity of genes involved in the differentiation of bASCs as well as a panel of surface markers to confirm the mesenchymal stem cell identity. The Aurum total RNA mini kit (Bio-Rad, Copenhagen, Denmark) was used to harvest total RNA from cultured cells. A nanoliter spectrophotometer (NanoDrop; Thermo Fisher Scientific, Wilmington, DE, USA) was used to determine RNA purity and concentration. The iScript cDNA synthesis kit (Bio-Rad, Copenhagen, Denmark) was used for the synthesis of complementary DNA (cDNA). Amplification reactions for each cDNA sample were performed in duplicate, in a final volume of 20 µl. Each reaction consisted of cDNA, IQ SYBR Green Supermix (Bio-Rad), and the target-specific primers (Table 1). The reaction was carried out on a QuantStudio™ 5 Real-Time PCR System (Thermo Fisher Scientific, Wilmington, DE, USA). The reaction was carried out for 40 amplification cycles: DNA denaturation was performed at 95 °C for 3 min, 95 °C for 10 s, and annealing and extension were performed at the corresponding annealing temperature for 30 s. One housekeeping gene (Ubiquitously Expressed Prefoldin Like Chaperone, *UXT)* was used to obtain the relative expression level of each gene in different samples (Table 1).

**Table 1:** Genes, primer sequences, and annealing temperatures used in real time qRT-PCR analysis.

Gene symbol (protein symbol)	NCBI reference sequence	Forward primer sequence	Reverse primer sequence	Annealing temperature
*PPAR-γ* (PPARG)	NM_181024.2	5′-ATG-GAT-GAC- CAC-TCC-CAT- GCC-3’	5′-ATC-TGC-AAC- CAT-CGG-GTC- AGC-3′	61 °C
*FABP4* (FABP4)	NM_174314.2	5’-AGC-TGC-ACT- TCT-TTC-TCA- CCT-TGA-A-3’	5’-TTG-GCC-ATG- CCA-GCC-AGC- CAC-TTT-3’	64 °C
*UXT* (UXT)	NM_001037471.2	5’-CAG-CTG-GCC- AAA-TAC-CTT- CAA-3’	5’- GTG-TCT-GGG- ACC-ACT-GTG- TCA-A-3’	60 °C
*ITGB1* (CD29)	NM_174368.3	5’- CGC-CGC- GGG-AGA-AGA- TGA-AT-3’	5’- CCC-ACA-TGA- TTT-GGC-ATT- TGC-TTT-C-3’	63 °C
*CD44* (CD44)	NM_174013.3	5’- ATA-CCT-CGG- ATA-CCA-GAG- ACT-ACG-G-3’	5’- TTC-CGC-ATA- GGA-CCT-GAG- GTT-G-3’	64 °C
*PTPRC* (CD45)	NM_001206523.1	5’-CTT-CTG-GCA- TTC-GGC-TTT- GCC-3’	5’- AAC-GCT-GGA- TGC-AGT-GGT- CA-3’	63 °C
*5NTD* (CD73)	NM_174129.4	5’- ACA-GGC- GAG-TTT-CTG- CAG-GT-3’	5’- AGA-GGC-TCA- TAA-CTG-GGC- ACA-C-3’	63 °C
*ALCAM* (CD166)	NM_174238.1	5’- CAG-CTG- GAG-AGA-ACA- GTA-AAC-TCC-T-3’	5’- AGG-AGA-CCA- ACA-ACA-ATT- CCC-ACA-A-3’	64 °C

### Visualization of intracellular lipid accumulation

Intracellular lipid droplets were visualized using both colorimetric and fluorometric assays. Cells were first fixed with 4% paraformaldehyde (AppliChem, Esbjerg, Denmark) in PBS for 10 min. Following fixation, for the colorimetric visualization, the cells were washed once with PBS and then stained with a 0.5% Oil Red O solution in ethanol for 30 min. Brightfield images were captured using an Axio-Observer Z1 microscope (Carl Zeiss) equipped with a digital camera (C11550 ORCA, Hamamatsu) and processed using ZEN 2012 software (Carl Zeiss). For fluorometric visualization, cells were incubated with 1 µg/ml BODIPY 493/503 in DMSO (Sigma-Aldrich) for 30 min at 37 °C. Confocal images were acquired using an LSM 900 confocal laser scanning microscope (Carl Zeiss) with an Airyscan detector and subsequently processed with ZEN 3.4 software (Carl Zeiss).

### Evaluation of proliferation potential

To evaluate the maintenance of proliferation potential after passaging, cells previously cultured in each medium until passage 9 were seeded in triplicate into 6-well plates. After 24 h of culture, cells were fixed with 4% paraformaldehyde for 15 min at room temperature followed by permeabilization with 0.1% Triton X-100 in PBS for 5 min. Nonspecific binding was blocked with 1% bovine serum albumin (BSA) in PBS for 30 min. The cells were then incubated with a polyclonal rabbit anti-Ki-67 primary antibody (PA5-19462, Invitrogen, dilution 1:100). After washing, the cells were incubated with a goat anti-rabbit IgG (H + L) antibody conjugated with Alexa Fluor 555 (Invitrogen, dilution 1:250). The primary and secondary antibodies were incubated for 1 h at room temperature with gentle shaking. The cell nuclei were counterstained with Hoechst 33,342 (Sigma-Aldrich, dilution 1:1000). Samples were observed using a fluorescence microscope (Axio Observer Z1, Carl Zeiss) and images were captured using a digital camera (C11440 ORCA Hamamatsu) and ZEN 2012 software. For each condition, six randomly selected fields were imaged at low magnification (5× objective) and the ZEN software’s built-in routines for automatic cell counting were used. The percentage of Ki-67–positive cells was calculated by dividing the number of Ki-67–positive nuclei by the total number of nuclei per field.

### Assessment of multipotency

Osteogenesis and adipogenesis assays were used to assess the maintenance of multipotency after passaging. The bASCs at passage 9 were seeded in triplicate in 24-well plates in serum-containing GM at a density of 8,000 cells/cm^2^. After reaching 80–90% confluence, the medium was replaced with induction medium. One group of wells was kept in GM as a negative control. For the osteogenesis assay, cells were induced with StemPro Osteogenic Differentiation Media (StemPro, ThermoFisher Scientific). After 8 days, cells were fixed with 4% formaldehyde for 1 h and the mineralized matrix was stained with Alizarin Red S (14 mg/ml) for 3 min. Cell images were taken under a standard inverted microscope (CKX41, Olympus). For the adipogenesis assay, cells were induced in two steps, as described in the main text: DM1 for 48 h and DM2 for 12 days. After washing the cells with PBS, lipid droplets were stained using Nile Red and nuclei were counterstained with Hoechst 33,342. Cells were incubated in Nile Red staining solution (2 µg/mL in PBS) at 37 °C in the dark for 30 min. Hoechst 33,342 (1:2000 dilution in PBS) was added and incubated for 15 min at room temperature in the dark. Following staining, wells were washed with PBS. Image acquisition was performed using the same microscope and software described for the cell viability assessment (Axio-Observer Z1 microscope and ZEN 2012 software).

### Statistical analysis

Statistical analyses were performed with GraphPad Prism 9 (San Diego, USA). The normality of the data distribution was assessed using the Shapiro-Wilk test. For comparisons between two groups, an independent samples t-test was used. When more than two conditions were compared, one-way analysis of variance (ANOVA) was performed, followed by Tukey’s post hoc test to assess differences between groups. Results are presented as mean ± standard error of the mean (SEM), with a p-value of < 0.05 considered statistically significant.

## Supplementary Information

Below is the link to the electronic supplementary material.

Supplementary Material 1

## Data Availability

The data generated during the current study are available from the corresponding author on reasonable request.
