# Supplementary material for: Development of a sustainable strategy for cultured fat production based on serum-free 3D culture of bovine adipose stem cells
Source: Sci Rep. 2025 Dec 29;15:44793. doi: 10.1038/s41598-025-28441-4 (PMC12749105; doi:10.1038/s41598-025-28441-4)
Supplement: Supplementary file 1 — Supplementary Material 1 [file 41598_2025_28441_MOESM1_ESM.docx]

**Development of a sustainable strategy for cultured fat production based on serum-free 3D culture of bovine adipose stem cells**

**Supplementary information file**


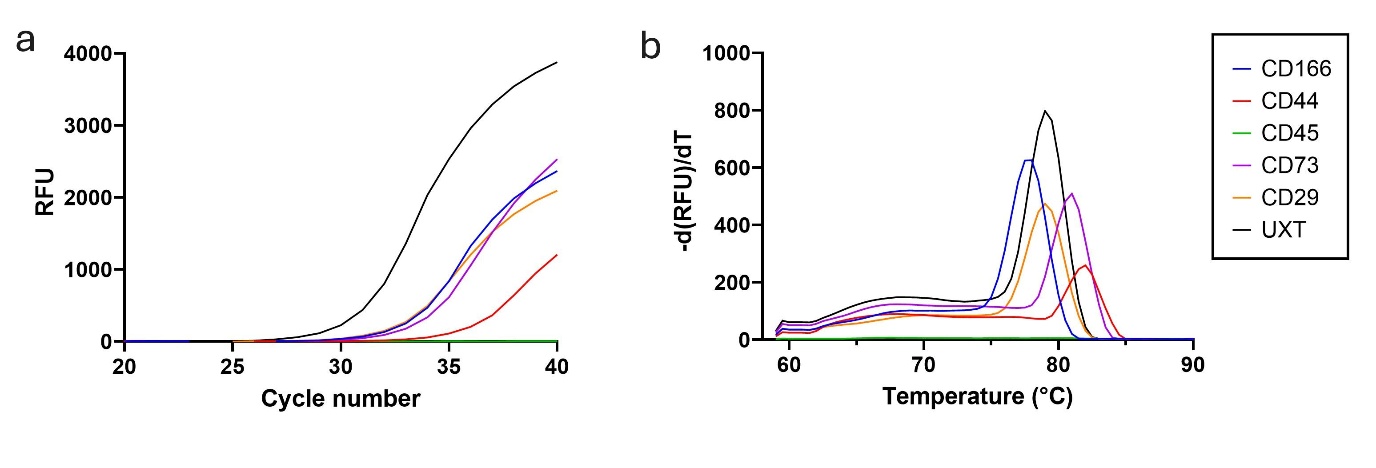


**Fig. S1.** Transcriptional profiling of surface markers in bovine adipose-derived stem cells (bASCs). (a) Real-time PCR amplification curves showing fluorescence intensity across cycles for *ALCAM* (CD166, blue), *CD44* (CD44, red), *PTPRC* (CD45, green), 5NTD (CD73, purple), *ITGB1* (CD29, orange), and the control gene *UXT* (UXT, black). *PTPRC* signal (CD45) was below detection limits. (b) Melt curve analysis confirming amplification specificity, with single peaks corresponding to each primer pair and no evidence of non-specific products or primer-dimer formation. No PCR product was detected for *PTPRC* (CD45).

**
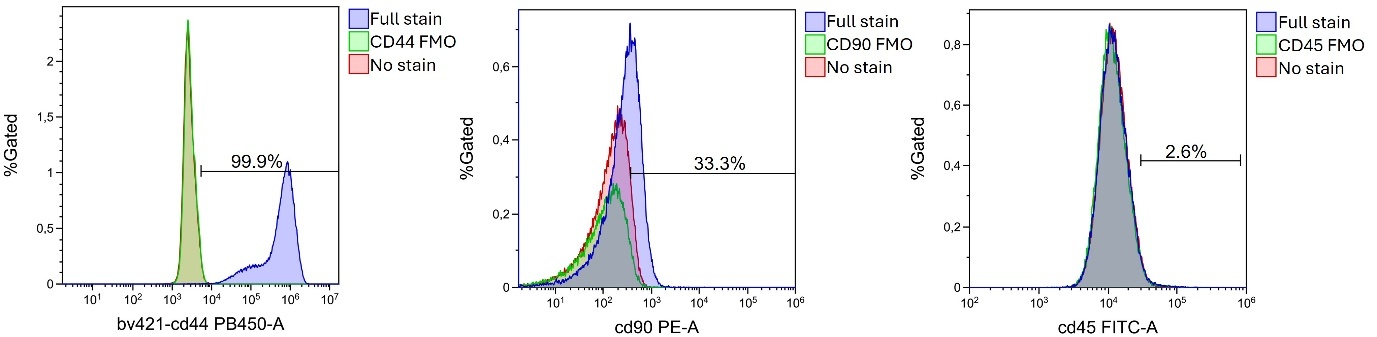
**

**Fig. S2.** Flow cytometric analysis of surface marker expression in bovine adipose-derived stem cells (bASCs). Histograms show fluorescence intensity profiles for CD44 (BV421), CD90 (PE), and CD45 (FITC). Each plot includes overlays for fully stained cells (blue), fluorescence minus one (FMO) controls (green), and unstained controls (red).


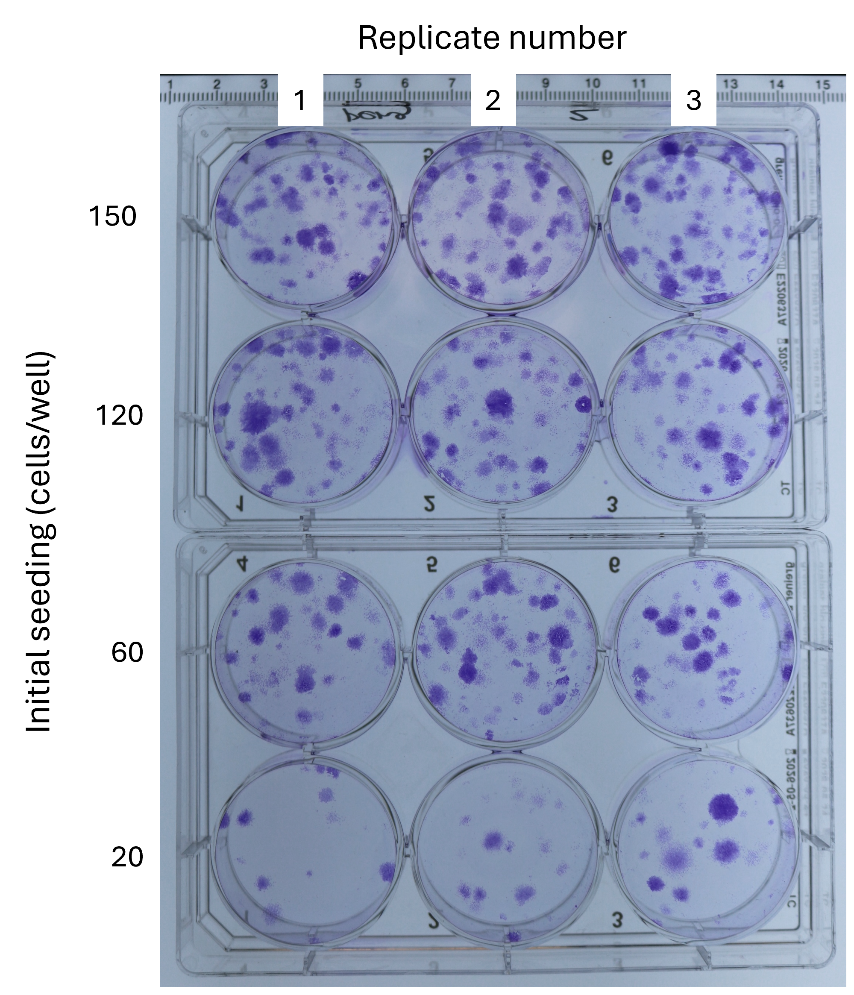


**Fig. S3.** Image of the plates displaying colonies formed after 14 days of culture. In the 60 cells/well group, the average number of colonies was 16.3 ± 2.4, resulting in an overall CFU-F efficiency of 27.3 ± 3.9%. The plates were flipped to better visualize the cell colonies in the wells (1, 2, and 3 indicate replicate number).


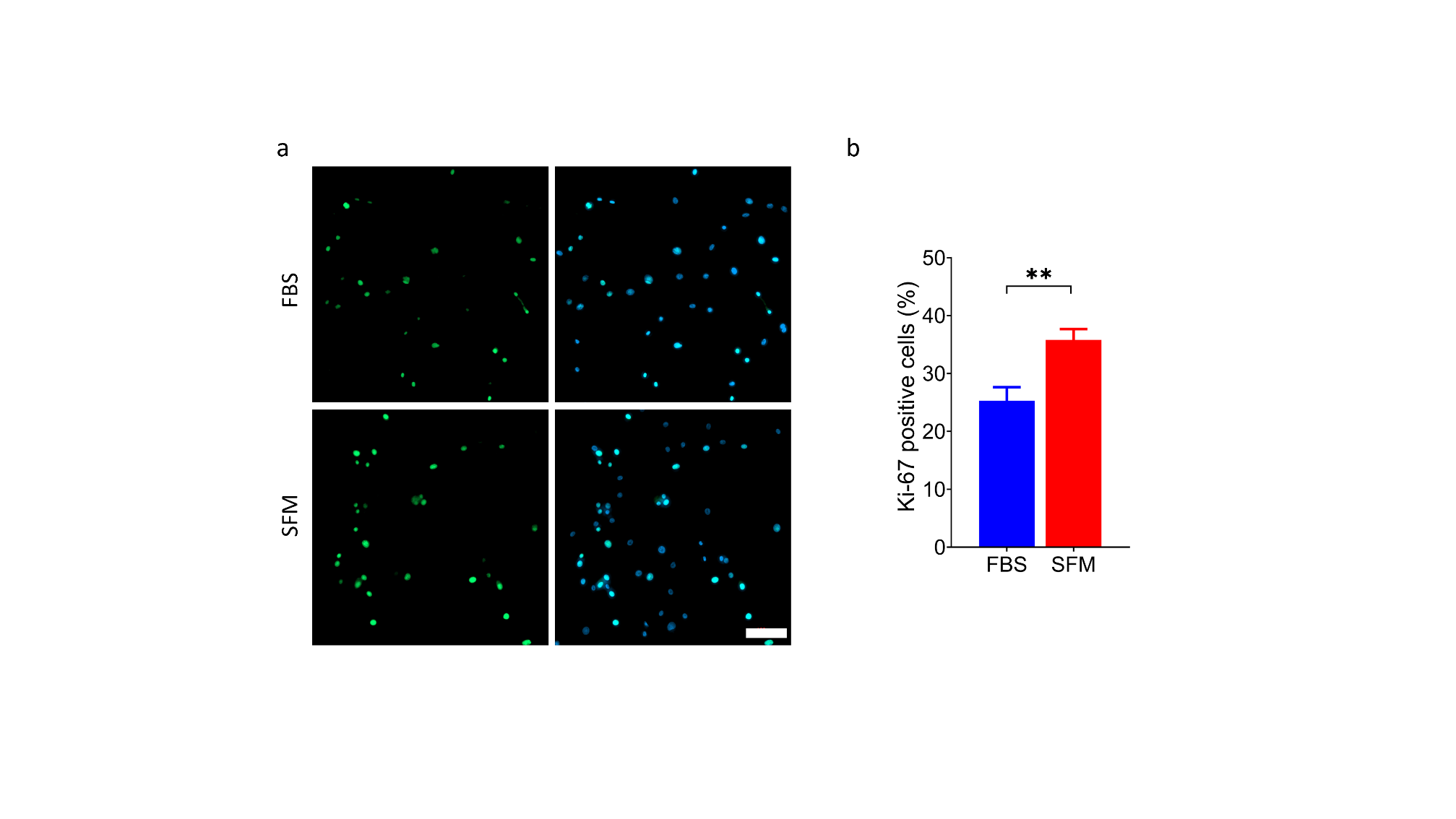


**Fig. S4.** Analysis of proliferation based on Ki-67 expression. (a) Representative images of Ki-67 staining (green) in bASCs grown in growth medium supplemented with fetal bovine serum (FBS) or serum-free supplement (SFM). The images on the right include Hoechst 33342 staining for the cell nuclei (blue). Scale bar = 100 µm. (b) Quantitative evaluation of the number of Ki-67-positive cells under the different culture conditions. (***p*<0.01, n=6).


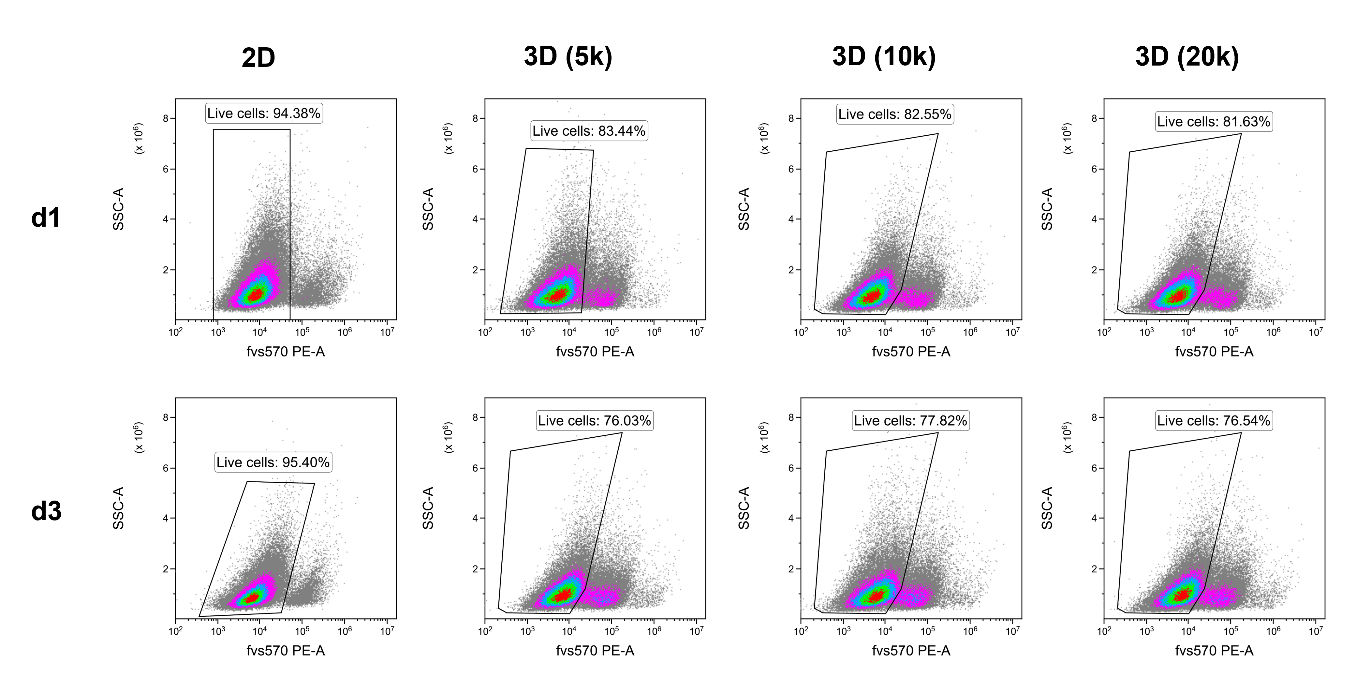


**Fig. S5.** Representative flow cytometry plots of live/dead cell staining in 2D and 3D cultures at day 1 (d1) and day 3 (d3). Cells were stained with the fixable viability dye 570 (FVS 570) and analyzed by flow cytometry. The x-axis represents fluorescence intensity, and the y-axis represents side scatter (SSC-A). Each plot shows the percentage of live cells within the gated population. Conditions include 2D culture and 3D spheroids seeded with 5,000, 10,000, or 20,000 cells.


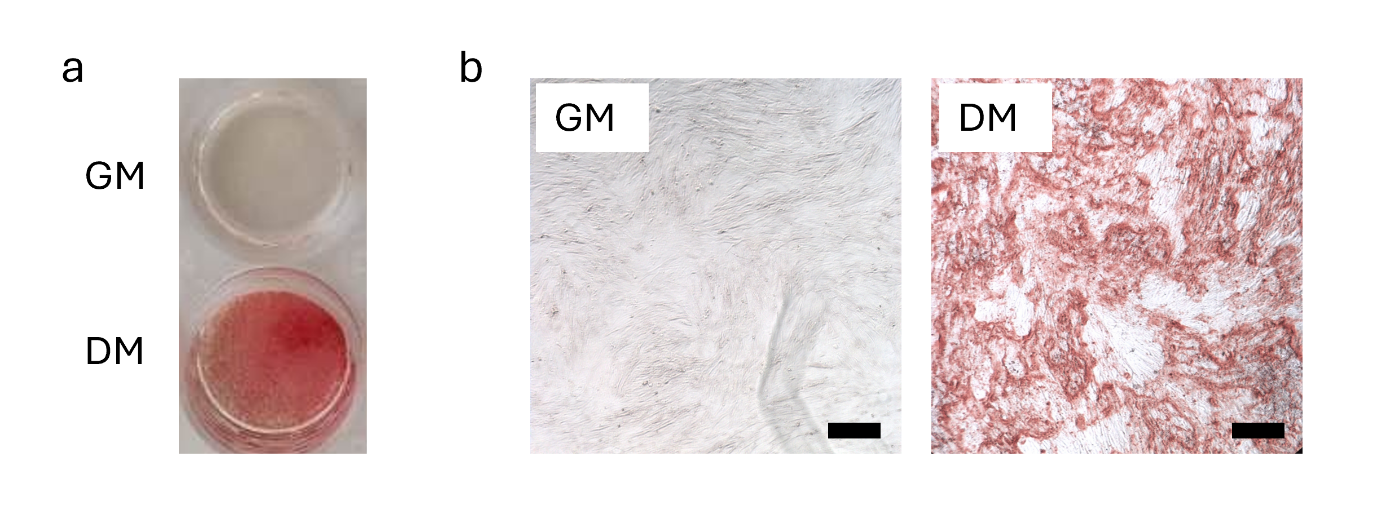


**Fig. S6.** Alizarin red staining of bASCs after osteogenic induction. (a) Image of the whole wells to visualize matrix production in cells cultured in osteogenic differentiation medium (DM) compared to cells cultured in growth medium (GM). (b) Phase contrast micrographs showing the mineral matrix secreted by the cells induced in DM as opposed to cells in GM. Scale bar = 100 µm.

**
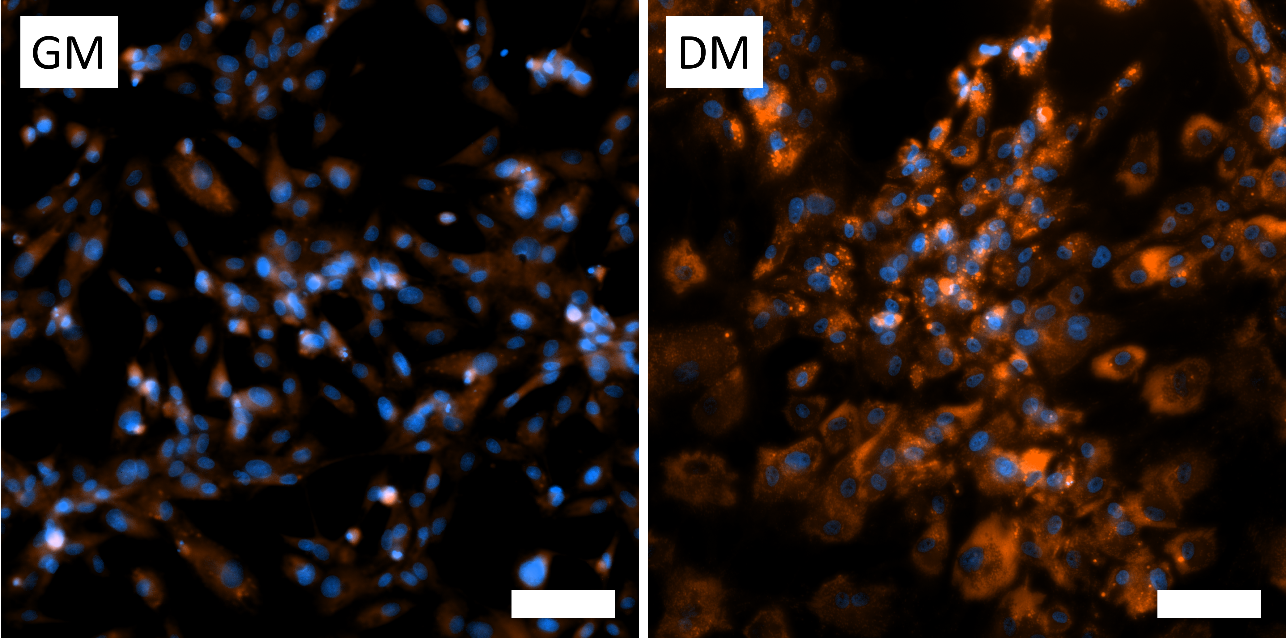
**

**Fig. S7.** Fluorescent microscopy images of bASCs cultured in growth medium (GM, left) and adipogenic differentiation medium (DM, right) for 12 days. Cells were stained with Nile Red (orange) to visualize lipid droplets and Hoechst 33342 (blue) to label nuclei. Scale bar: 100 µm.
